# High ectoine production by an engineered *Halomonas hydrothermalis* Y2 in a reduced salinity medium

**DOI:** 10.1186/s12934-019-1230-x

**Published:** 2019-10-26

**Authors:** Qi Zhao, Shannan Li, Peiwen Lv, Simian Sun, Cuiqing Ma, Ping Xu, Haijun Su, Chunyu Yang

**Affiliations:** 1 State Key Laboratory of Microbial Technology, Shandong University, Qingdao, 266237 People’s Republic of China; 2 School of Mathematics, Shandong University, Jinan, 250100 People’s Republic of China

**Keywords:** Ectoine synthesis, Doe pathway, Na^+^/H^+^ antiporter, Mrp antiporter, Reduced salinity medium

## Abstract

**Background:**

As an attracted compatible solute, 1,4,5,6-tetrahydro-2-methyl-4-pyrimidinecarboxylic acid (ectoine) showed great potentials in various field. However, lower productivity and high saline medium seriously hinder its wide applications.

**Results:**

The entire ectoine metabolism, including pathways for ectoine synthesis and catabolism, was identified in the genome of an ectoine-excreting strain *Halomonas hydrothermalis* Y2. By in-frame deletion of genes encoding ectoine hydroxylase (EctD) and (or) ectoine hydrolase (DoeA) that responsible for ectoine catabolism, the pathways for ectoine utilization were disrupted and resulted in an obviously enhanced productivity. Using an optimized medium containing 100 g L^−1^ NaCl in a 500-mL flask, the double mutant of Y2/*ΔectD*/*ΔdoeA* synthesized 3.13 g L^−1^ ectoine after 30 h cultivation. This is much higher than that of the wild type strain (1.91 g L^−1^), and also exceeds the production of Y2/*ΔectD* (2.21 g L^−1^). The remarkably enhanced accumulation of ectoine by Y2/*ΔectD*/*ΔdoeA* implied a critical function of Doe pathway in the ectoine catabolism. Furthermore, to reduce the salinity of fermentation medium and overcome the wastewater treatment difficulty, mutants that lacking key Na^+^/H^+^ antiporter, Mrp and (or) NhaD2, were constructed based on strain Y2/*ΔectD*/*ΔdoeA*. As a result, the Mrp-deficient strain could synthesize equal amount of ectoine (around 7 g L^−1^ or 500 mg (g DCW) ^−1^) in the medium containing lower concentration of NaCl. During a fed-batch fermentation process with 60 g L^−1^ NaCl stress, a maximum 10.5 g L^−1^ ectoine was accumulated by the Mrp-deficient strain, with a specific production of 765 mg (g DCW)^−1^ and a yield of 0.21 g g^−1^ monosodium glutamate.

**Conclusion:**

The remarkably enhanced production of ectoine by Y2/*ΔectD*/*ΔdoeA* implied the critical function of Doe pathway in the ectoine catabolism. Moreover, the reduced salinity requirement of Mrp-deficient strain implied a feasible protocol for many compatible solute biosynthesis, i.e., by silencing some Na^+^/H^+^ antiporters in their halophilic producers and thus lowering the medium salinity.
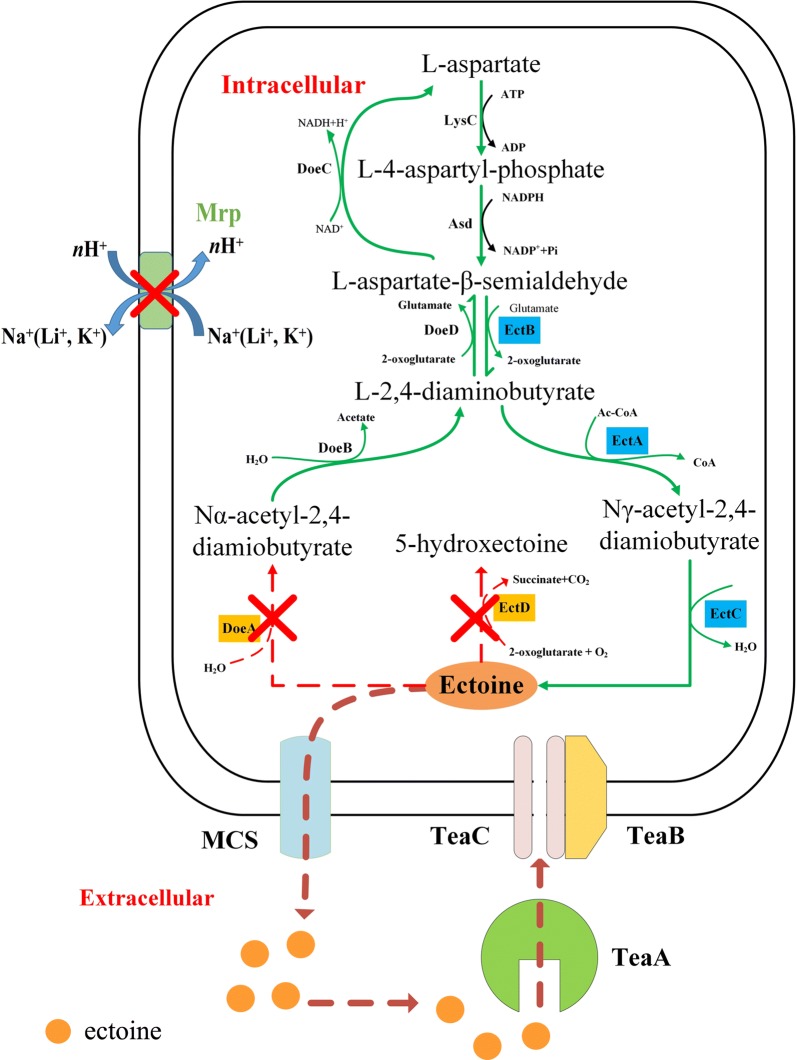

## Background

Ectoine (1,4,5,6-tetrahydro-2-methyl-4-pyrimidinecarboxylic acid), a cyclic amino acid derivative, is one of the most widely compatible solutes throughout some halophilic and halotolerant microorganisms, as well as a dominant compatible solute in halophilic methanotrophs and methylotrophs [1–4]. Besides its primary function of protecting cells against high osmolarity, the most powerful stabilizing properties on biological macromolecule (enzymes, DNA, antibodies, and even whole cells) confer ectoine attractive potentials in fields of skin caring, food processing, molecular biology, agriculture, biotechnology, and medical values in human diseases [5–11].

Ectoine can be synthesized by a large number of aerobic, heterotrophic bacteria, such as genus *Halorhodospira*, genera *Halomonas, Chromohalobacter, Vibrio, Pseudomonas* of the class γ-*proteobacteria*, and even archaea *Nitrosopumilus maritimus* or methanotroph *Methylomicrobium alcaliphilum* strain [12–15]. Commercially, it is produced by the moderate halophilic bacterium *Halomonas elongata*, which has an established biosynthetic pathway for ectoine metabolism. Starting from precursor l-aspartate-β-semialdehyde (ASA), ectoine is synthesized by the catalytic combination of a diaminobutyric acid transaminase (EctB), a diaminobutyric acid acetyltransferase (EctA), and an ectoine synthase (EctC) [16, 17]. Under certain stress conditions (e.g. elevated temperatures), it is demonstrated that some ectoine could be converted to 5-hydroxyectoine by ectoine hydroxylase (EctD) [18, 19].

In the microorganisms, ectoine and 5-hydroxyectoine are not only accumulated as excellent stress protectants but can also serve as valuable nutrients for cell growth [20]. Similar gene clusters involving in ectoine catabolism were disclosed in strain *Sinorhizobium meliloti* [21], *Ruegeria pomeroyi* DSS-3 [22], and *H. elongata* DSM 2581^T^ [23]. In *H. elongata* DSM 2581^T^, the gene cluster comprising of *doeA* (ectoine hydrolase), *doeB* (Na-acetyl-l-2,4-diaminobutyric acid deacetylase), *doeC* (aspartate-semialdehyde dehydrogenase), and *doeD* (diaminobutyric acid transaminase) was verified by in-frame deletion. Recently, more detailed catabolic pathway, as well as its regulatory system was identified in strain *R. pomeroyi* DSS-3 [24, 25]. These genetic circuit(s) suggest avenues for the genetic controlling of ectoine accumulation and are valuable for ectoine production.

Since hyper-osmolarity pressure is required for compatible solutes accumulation, fermentation medium with high salinity is generally used for their enrichments and raise big challenges for the fermenter antirust and wastewater treatment. Therefore, low-salinity medium is desirable for large-scale ectoine production, as well as for some other compatible solutes. *H. hydrothermalis* Y2 was isolated from an artificial alkaline environment of pulp mill wastewater. As a halotolerant extremophile, the strain grows well in a broad range salinity that from 0 to 180 g L^−1^ NaCl [26]. As we previously observed, four Na^+^/H^+^ antiporters work in a labor division way to deal with saline and alkaline environments, in which NhaD2 and Mrp play a notable physiological role in pH and osmotic homeostasis [27]. In the present study, based on a double mutant that lacking *doeA* and *ectD* genes, Mrp and (or) NhaD2 were in-frame deleted and their effluence to the ectoine productivity was investigated.

## Results

### Predicted ectoine metabolic pathway in *H. hydrothermalis* Y2

**Fig. 1:**
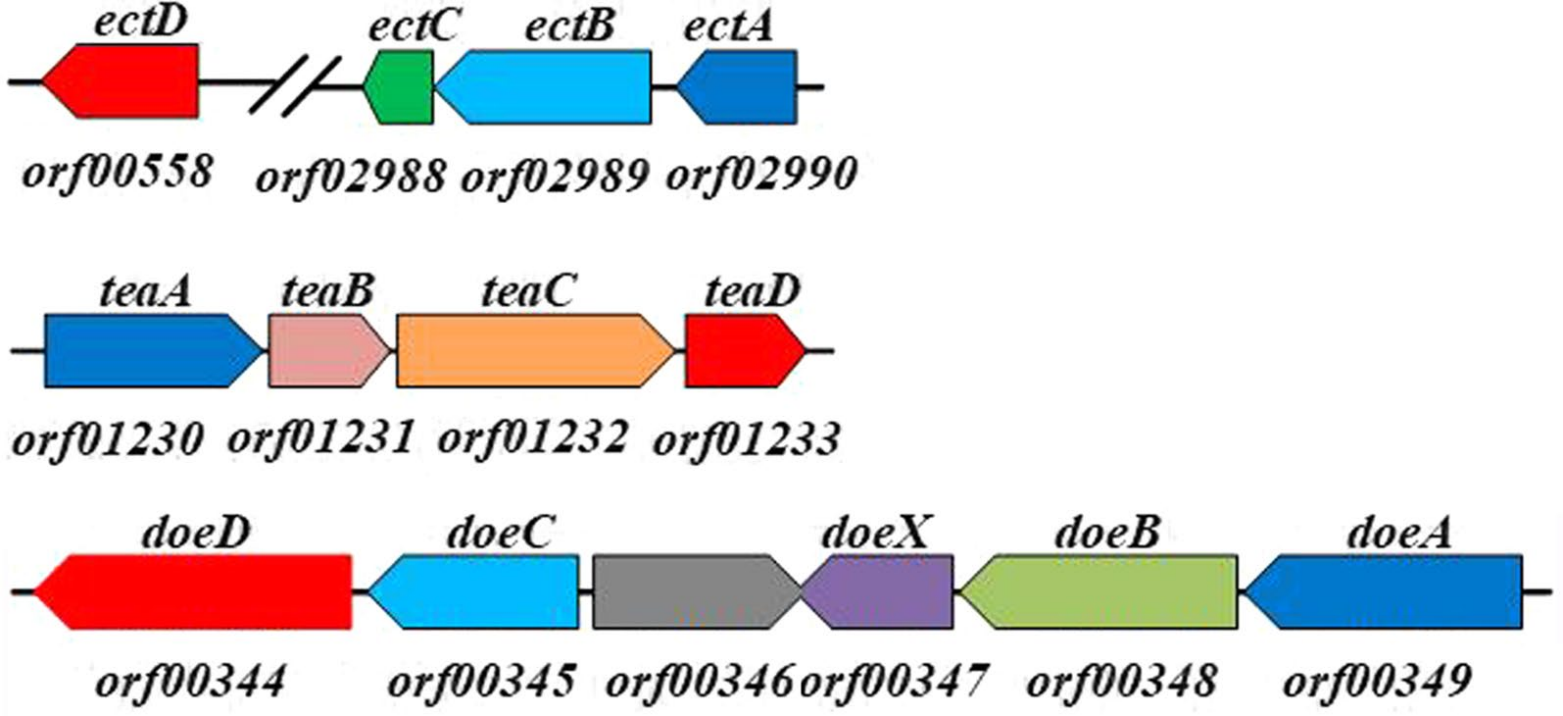
Predicted gene clusters for the metabolic pathways of ectoine in strain *H. hydrothermalis* Y2. *ectA*: l-2,4-diaminobutyric acid Nγ-acetyltransferase gene (No. ATH78870.1); *ectB*: l-2,4-diaminobutyric acid transaminase gene (No. ATH78869.1); *ectC*: ectoine synthase gene (No. ATH78868.1); *ectD*: ectoine hydroxylase gene (No. ATH76535.1); *doeA*: ectoine hydrolase gene (No. ATH76416.1); *doeB*: Na-acetyl-l-2,4-diaminobutyric acid deacetylase gene (No. ATH76415.1); *doeX*: transcriptional regulator (No. ATH76414.1); *doeC*: aspartate-semialdehyde dehydrogenase gene (No. ATH76412.1); *doeD:* l-2,4-diaminobutyric acid transaminase gene (No. TH76411.1); *teaA*: ectoine-binding periplasmic protein gene (No. ATH77233.1); *teaB*: ectoine TRAP transporter small permease gene (No. ATH77234.1); *teaC*: ectoine TRAP transporter large permease gene (No. ATH77235.1); *teaD*: TRAP-T-associated universal stress protein encoding gene (No. ATH77236.1)

As observed in the genomes of *H. elongata* DSM 2581 ^T^ and *Chromohalobacter salexigens* [23], two gene clusters which is localized in the chromosome of *H. hydrothermalis* Y2 (NCBI no: CP023656) are potentially involved in ectoine synthesis and catabolism (Fig. 1). Enzymes for the biosynthesis pathway, i.e., EctA (Orf02990), EctB (Orf02889), and EctC (Orf02888) are clustered together. In addition, ectoine hydroxylase (EctD) encoding gene which responsible for 5-hydroxyectoine synthesis (*Orf00558*) is also present in the genome but locates distantly. The pathway for ectoine degradation locates in another cluster which comprises of six orfs ranging from *Orf00344* to *Orf00349*, encoding putative enzymes of DoeA, DoeB, DoeX, DoeC, and DoeD, respectively. These enzymes exhibited > 80% protein identity with those of *H. elongata* DSM2581^T^. As depicted in the ectoine model of *H. elongata* DSM2581^T^, these enzymes form a metabolic cycle for ectoine production and degradation [23]. Compared to *H. elongata* DSM2581^T^, an additional ORF containing 406 aa (*Orf00346*) locates before the *doeX* gene (*Orf00345*). Sequence analysis in Universal Protein Resource (UniProt) and NCBI database (http://www.ncbi.nlm.nih.gov/) indicated a possibility that this putative protein is a diguanylate cyclase (GGDEF) domain that involving the synthesis of the important secondary massager of c-di-GMP [28, 29]. Based on the sequence blast in NCBI, we searched the genomes of reported *Halomonas* species and found only few *Halomonas* strain possessed this protein in the Doe pathway. Further detailed study needs to be addressed for the function of this putative protein in ectoine metabolism.

### Ectoine production by Doe pathway destructive strains

**Fig. 2:**
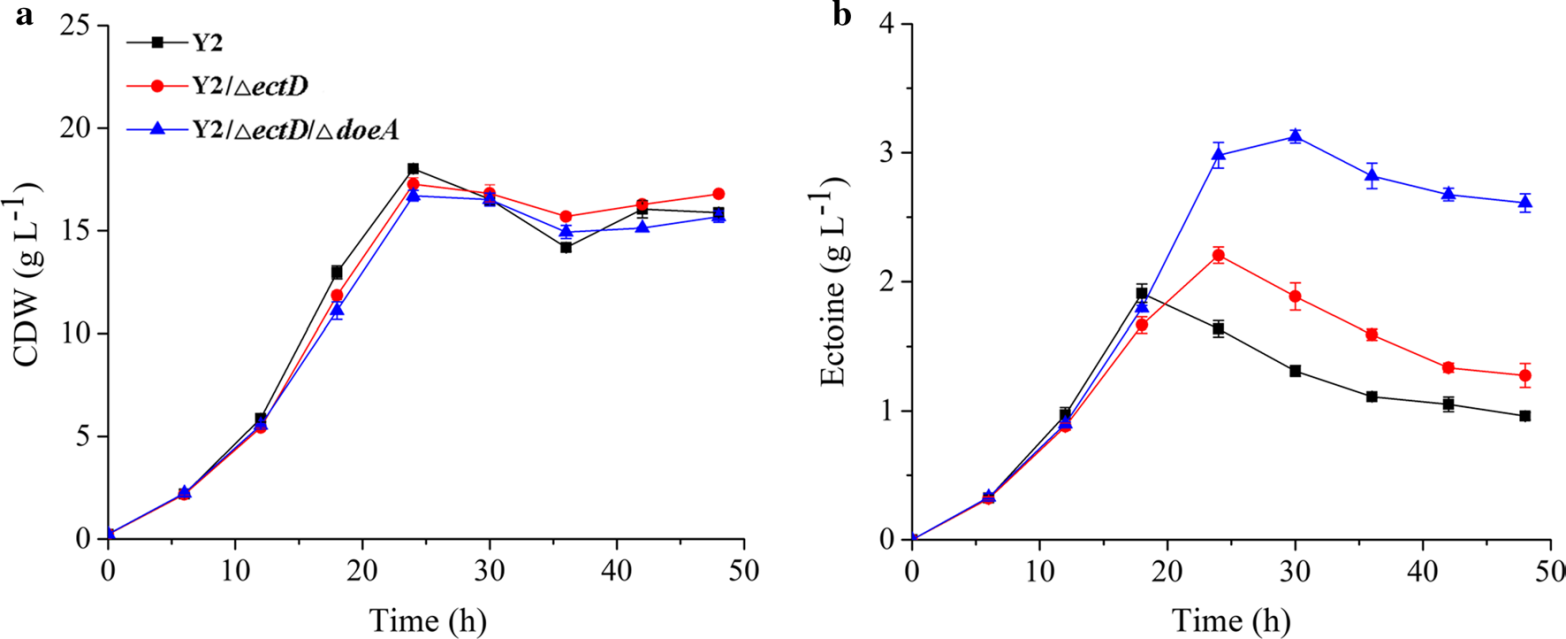
Growth and ectione production of strain *H. hydrothermalis* Y2, Y2/*ΔetcD*, and Y2/*ΔetcD*/*ΔdoeA* in 500-mL flask containing 100 g L^−1^ NaCl at pH 9.0. **a** Cell dry weight (CDW). **b** Total ectoine titer (g L^−1^). Each experiment was conducted in triplicate

In the predicted pathway of *H. hydrothermalis* Y2, ectoine can be utilized as the substrate for hydroectoine synthesis or cell nutrients, as that of observed in strain *H. elongata* DSM2581^T^. To block these ectoine consumptions and gain more ectoine accumulation, we knocked out gene of *ectD* and (or) *doeA* in the genome of Y2, and thereby constructed a single mutant Y2/*ΔectD* and double variant of Y2/*ΔectD/ΔdoeA*. By using MG medium [30] with some modifications (modified monosodium glutamate medium, MMG medium, pH 9.0) with 34 g L^−1^ monosodium glutamate (MSG) and 100 g L^−1^ NaCl composition, the growth of *H. hydrothermalis* Y2 and two mutants, as well as ectoine productions were compared in 500-mL flasks. It is worth mentioning that, high alkalinity resulted in a certain amount of precipitations in the MMG medium. Along with contributions of large doses MSG and NaCl in this ectoine-producing medium, a relative higher value of cell dry weight (CDW) were detected as shown in Fig. 2a, as well as all other fermentation batches in this study. Expectedly, the ectoine production of these three strains were gradually increased with the strain growth (Fig. 2b), the maximum ectoine titer for Y2 is 1.91 g L^−1^ at 18 h, while a maximum productivity of 2.21 and 3.13 g L^−1^ was obtained after 24, and 30 h cultivations by mutants Y2/*ΔetcD* and Y2/*ΔetcD*/*ΔdoeA*, respectively. As shown in Fig. 2a, both the wild type strain Y2 and Y2/*ΔectD* yielded a second growth after 30 h cultivation and simultaneously consumed the synthesized ectoine (Fig. 2b). After 48 h cultivation, totally 50% of produced ectoine was degraded by the wild type strain Y2. In compared with strain Y2, less ectoine was consumed by two mutants, especially by strain Y2/*ΔetcD*/*ΔdoeA* (20% degradation). We suspected the deficiency of these two catabolic pathways resulted in a continuously increase of ectoine after reaching to its maximum biomass, and thereby the ectoine accumulation exhibited prolonged curves than that of strain Y2. As a result, the double mutant Y2/*ΔectD/ΔdoeA* could synthesized 63.8% additional ectoine when compared to the wild type strain and the consumption ratio of the double mutant was lower than those of the other two strains. This implied the disruption of ectoine degradative pathway, especially the Doe pathway, was greatly contribute to ectoine accumulation in this strain. However, it is worth noting that around 20% ectoine was still depleted during the later stage of fermentation of the double mutant. This suggested that other unknown degradative pathway might be exist in the genome of strain Y2 and utilize ectoine as carbon or nitrogen source.

### Ectoine production by Na^+^/H^+^ antiporter deficient strains under various NaCl stress

**Fig. 3:**
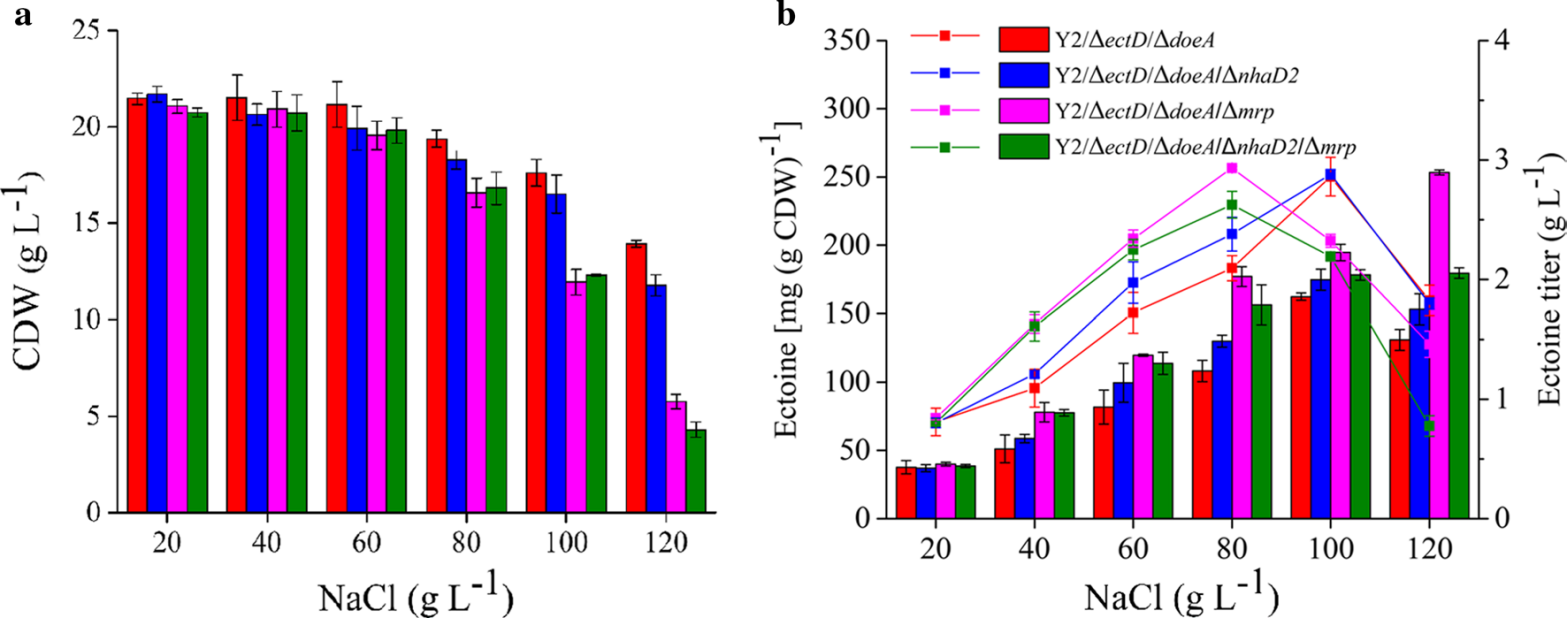
Growth (**a**) and ectoine production (**b**) of *H. hydrothermalis* Y2 and its mutants in various NaCl concentrations. Lines represent total ectoine titer (g L^−1^) and columns represent total ectoine specific production [mg (g CDW)^−1^]. The strains were cultured in 500-mL flask containing various NaCl at pH 9.0. Each experiment was conducted in triplicate

To alleviate the saline medium of the strain for ectoine synthesis, two major Na^+^/H^+^ antiporters in mutant Y2/*ΔectD/ΔdoeA* were deleted and tested for their abilities in ectoine accumulation in the 500-mL flasks. After 24 h cultivation, mutant Y2/*ΔectD/ΔdoeA* gained a maximum growth in the range of 20–60 g L^−1^ NaCl and then gradually inhibited by elevated salinity (Fig. 3a). Expectedly, high saline stress is essential to the ectoine accumulation, with the highest productivity was detected upon 100 g L^−1^ NaCl stress (Fig. 3b). The absence of NhaD2 antiporter slightly decreased the cell growth than that of Y2/*ΔectD/ΔdoeA*, while the ectoine synthesis displayed a similar profile under lower salinity (< 80 g L^−1^) and a maximum content was also detected in the medium with 100 g L^−1^ NaCl addition. Differently, the deletion of Mrp antiporter greatly affected the strain growth under high salinity and thus resulted in a high specific values. As shown in Fig. 3a, the growth of *mrp*-deficient strains was obviously inhibited by high NaCl stress, especially in the medium containing NaCl higher than 100 g L^−1^. Albeit of the impaired growth, the *mrp* deficiency led to an obviously enhanced synthesis of ectoine under lower salinity (Fig. 3b), with a maximum titer observed in media containing 80 g L^−1^ NaCl. In the medium with 80 g L^−1^ NaCl supplementation, the ectoine production (2.93 g L^−1^) of Y2/*ΔectD/ΔdoeA/Δmrp* was equal or even slightly higher than the maximal production (2.86 g L^−1^) of strain Y2/*ΔectD/ΔdoeA* which accumulated in the medium containing 100 g L^−1^ NaCl. Under higher saline stress, the absence of Mrp seriously impaired the cell growth and the ectoine titer decreased drastically. However, the mutated cells were more sensitive to high saline environments (120 g L^−1^ NaCl) and simultaneously synthesized more ectoine for protecting the cells. As a result, a significant increase of the specific ectoine production was observed by mutants lacking Mrp antiporters (Fig. 3b).

### Batch fermentation

**Fig. 4:**
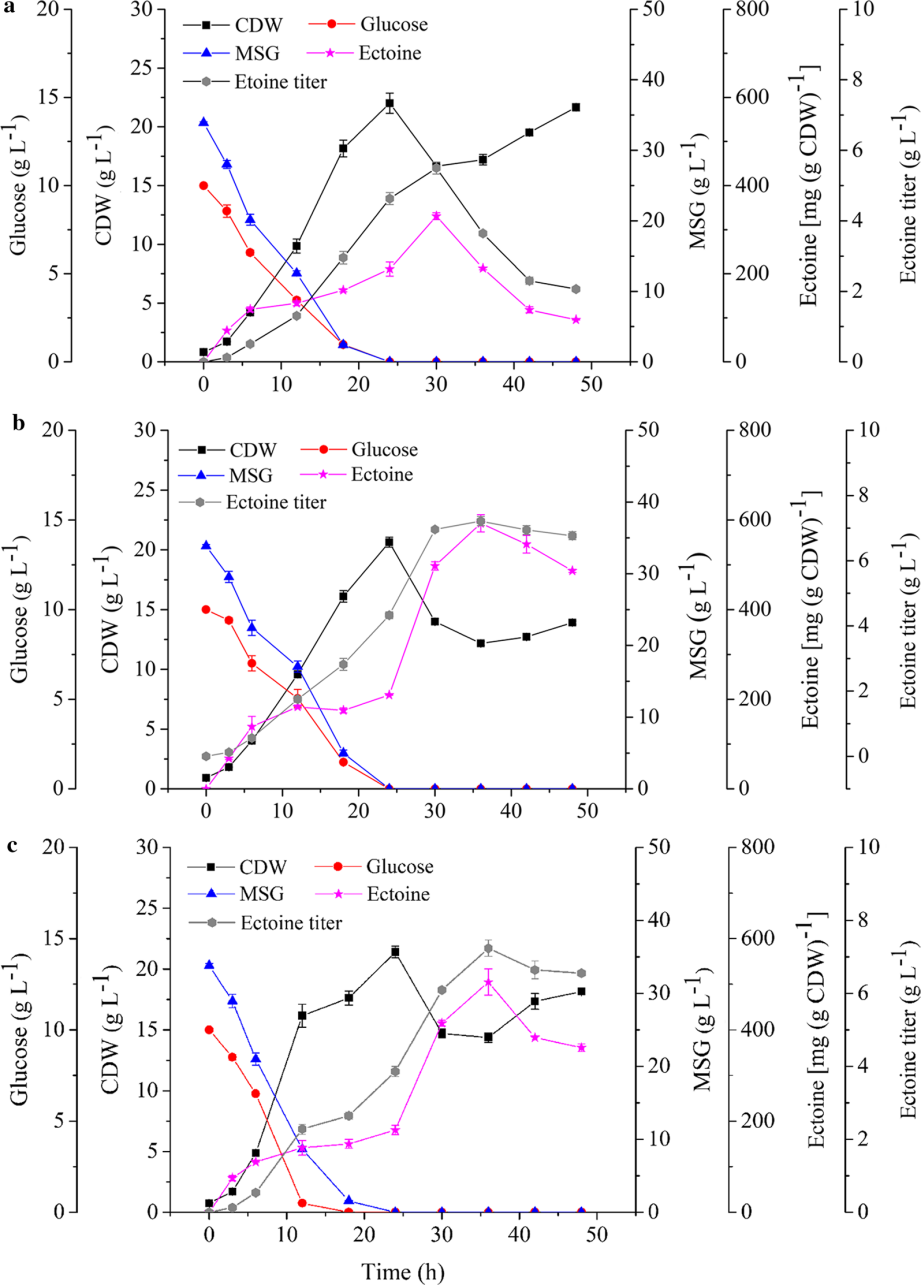
Batch fermentation of *H. hydrothermalis* Y2 (**a**), Y2/*ΔetcD*/*ΔdoeA* (**b**) and Y2/*ΔetcD*/*ΔdoeA/Δmrp* (**c**) mutants in the 1-L auto-controlled fermenter and growth of three strains in the MMG medium at pH 9.0. Each time was sampled in duplicate

The ectoine-producing capacity of Y2, Y2/*ΔectD/ΔdoeA*, and Y2/*ΔectD/ΔdoeA/Δmrp* were further compared in the 1-L bioreactor which equally controlled, by keeping same inoculation and culture conditions. During 48 h cultivation, these three strains exhibited a similar growth profile in the first cultivation stage (Fig. 4a), by reaching to the stationary stage after 24 h incubation and all displayed a secondary growth after 30 h. Compared to its mutants, strain Y2 yielded a vigorous growth in this secondary growth and correspondingly a remarkably decreased ectoine content was observed. As shown in Fig. 4a, the biomass (CDW) increased from 16.6 to 21.7 g L^−1^ during the cultivation period of 36–48 h. In the meantime, the total ectoine titer decreased from 5.5 to 2.0 g L^−1^ and the corresponding consumption rate of ectoine was 0.191. However, the reduction of ectoine contents of two mutants are slight, from 7.2 to 6.7 g L^−1^ in mutant Y2/*ΔectD/ΔdoeA* and 7.2 to 6.5 g L^−1^ in Y2/*ΔectD/ΔdoeA/Δmrp* (Fig. 4b, c). Consequently, the consumption rate of ectoine during this stage was much lower than that of the wild type strain (0.037 and 0.057, respectively), and the secondary growth of these two mutants were weak than that of the wild type strain, with a biomass increase from 12.1 to 13.9 g of Y2/*ΔectD/ΔdoeA* and 14.3 to 18.1 g of Y2/*ΔectD/ΔdoeA/Δmrp*. Notably, under lower saline stress (80 g L^−1^ NaCl), mutant Y2/*ΔectD/ΔdoeA/Δmrp* showed a rapid growth during the first 12 h incubation, along with a quickly consumption of glucose and MSG. This implied that lower NaCl is beneficial to the strain growth but pleasantly did not affect the ectoine productivity.

### Fed-batch fermentation of strain Y2/*ΔectD/ΔdoeA/Δmrp*

**Fig. 5:**
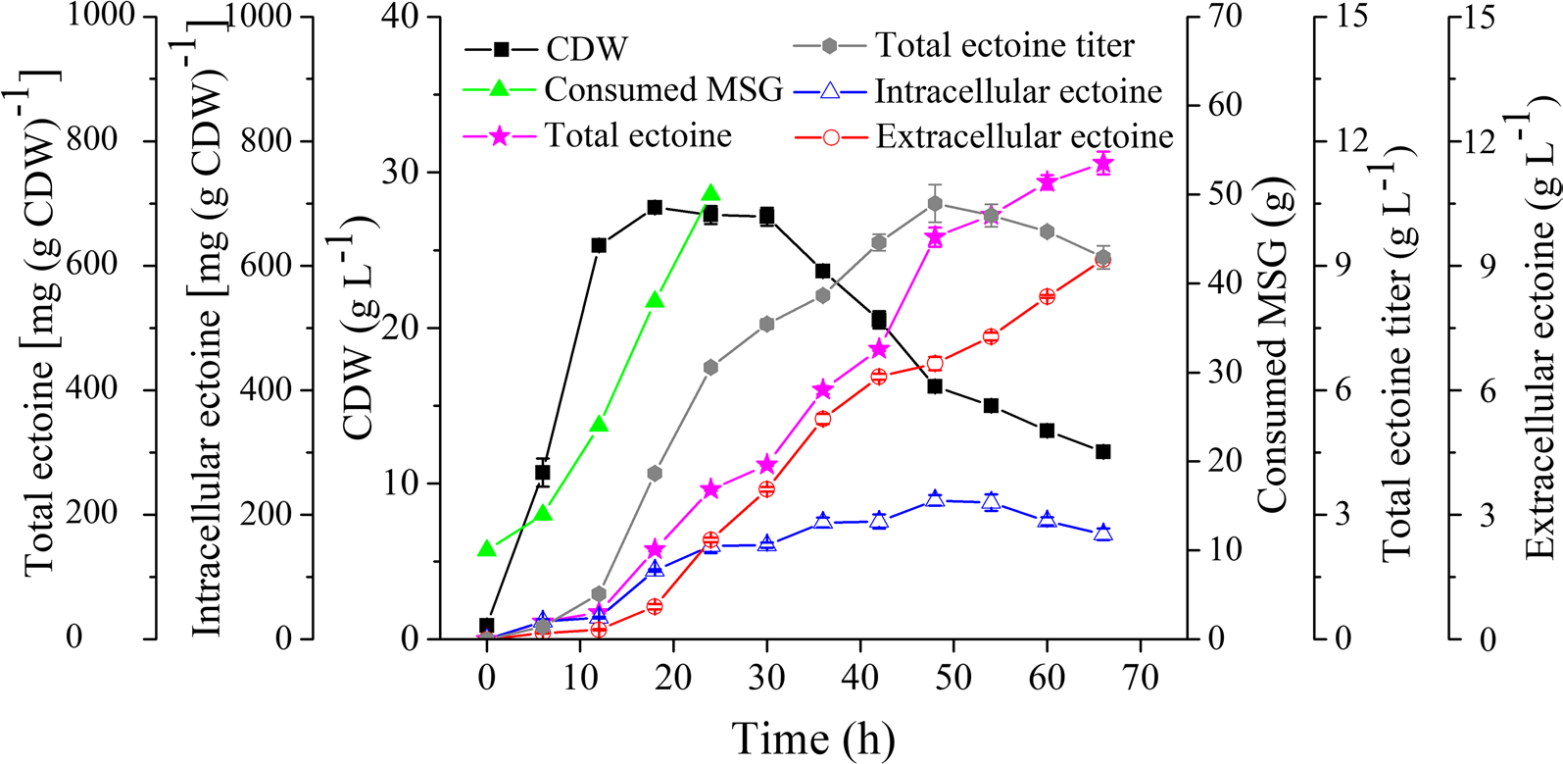
Fed-batch fermentation of Y2/*ΔetcD*/*ΔdoeA/Δmrp* in 1-L fermenter containing 800 mL MMG medium. During fermentation, NaCl was added from 20 g L^−1^ to 60 g L^−1^ and MSG were fed to keep a 20 g L^−1^ concentration. Each time was sampled in duplicate

To improve the ectoine production of Y2/*ΔectD/ΔdoeA/Δmrp*, the strain was cultured in a 1-L fermenter which containing 0.5 L of MMG medium and a fed-batch protocol was used. Based on our previous observation, strain Y2 is a halotolerant strain and grows best under a concentration of 20–30 g L^−1^ NaCl [26]. To get a higher cell biomass, the initial salinity of the medium was 20 g L^−1^ and increased to 60 g L^−1^ after 18 h incubation. As a result, the strain grew much quickly and more biomass was obtained, with a maximum CDW of 27.8 g L^−1^ after 18 h incubation (Fig. 5). This is obviously higher than the growth of mutant Y2/*ΔectD/ΔdoeA/Δmrp* that shown in Fig. 4c. Compared with the batch fermentation process, the cells retained a longer stationary phase and no second growth phase observed. As shown in Fig. 5, the whole fermentation progress could be divided into three phases. The strain grew fast in the first phase (0–18 h), along with a quickly accumulation of the ectoine contents in the cells and 0.21 g L^−1^ ectoine was detected in the medium. In the second phase (24–48 h), the strain entered stationary phase in which intracellular content of ectoine reached to its maximum 223.0 mg (g CDW)^−1^ and kept relatively constant. In the meanwhile, ectoine was gradually excreted and accumulated in the medium, and thus the total ectoine titer reached to a maximum value of 10.5 g L^−1^ after 48 h fermentation. In the third phase (48 to 72 h), the biomass exhibited a sharply decrease and intracellular ectoine content decreased simultaneously. We suspected that this reduction mainly derived from the cell autolysis and some ectoine released into the medium. Therefore, the total ectoine specific production kept increased to 765 mg (g CDW)^−1^ at the fermentation end-point. During the whole fermentation process, totally 50 g MSG was consumed and the overall ectoine yield from per gram MSG was 0.21 g.

## Discussion

Special repair and protective functions confer ectoine an attractive compatible solute for wide applications. Genome sequencing of those ectoine-producing strains provide more information to the metabolic engineering efforts for high ectoine productivity. Through a hydroxylation reaction mediated by the EctD enzyme, a member of the non-heme-containing iron (II) and 2-oxoglutarate-dependent dioxygenases, ectoine can be transformed to another osmotic solute of hydroectoine [31]. Together with ectoine, hydroxyectoine is often synthesized at lower amounts by many ectoine-producing species [2]. In an *H. elongata*-derived *etcD*-deficient strain, the ectoine production was 1.56-fold higher than that of the wild type strain [32]. Therefore, we first constructed an *etcD*-deficient strain from *H. hydrothermalis* Y2 and obtained a 1.16-fold ectoine yield relative to that of *H. hydrothermalis* Y2. This slightly enhanced ectoine production of Y2/*ΔetcD* suggests that hydroectoine is not the main compatible solute in this strain.

A metabolic cycle for ectoine synthesis and catabolism had been established by Schwibbert and his colleagues [23] in the genome of *H. elongata* DSM2581^T^. In strain *H. elongata* DSM2581^T^, the deletion of members in gene cluster *doeABCD* led to the inability of the mutants to utilize ectoine as a carbon source or a reducing growth on ectoine. Therefore, these genes were proposed to be responsible for the breakdown of ectoine into aspartate. In the genome of *H. hydrothermalis* Y2, a similar gene cluster encoding Doe proteins was also observed (Fig. 1). Expectedly, the deletion of *doeA* in this study resulted in a remarkably enhanced ectoine production. In the 500-mL flasks, the maximum intracellular ectoine of Y2/*ΔectD*/*ΔdoeA* was 1.64-fold higher than that of the wild type strain. In compared to the 1.16-fold ectoine increase of Y2/*ΔectD*, we proposed that this Doe pathway is critical to the ectoine utilization and is of great potential in metabolic engineering for high ectoine production. Especially, for those ectoine producers of *Proteobacteria* strains that also carrying at least two *doeA* genes [23]. Apart of the decreased ectoine consumption and enhanced productivity, we also noticed that only around 80% of produced ectoine was retained in the double mutant, implying that some ectoine was consumed by the cells and some other pathways for ectoine utilization might be exist in this strain that need to be further addressed.

Besides of those genetic engineering efforts for high ectoine productivity, various medium optimization efforts and technology strategies were also employed [33–35]. By applying isotope label tracing, Pastor and coworkers [36] have revealed that the metabolism of *C. salexigens* is adapted to support high metabolic fluxes in high saline environments, and towards a synthesis of its major compatible solute of ectoine. In a recently reported strain *Halomonas* sp. SBS 10, the *de*-*novo* synthesis of ectoine also significantly increased under high osmotic stress of 25% NaCl [37]. In agreement with these halotolerant and halophilic ectoine-producers [33, 38–40], NaCl was also screened to be a key factor on the ectoine synthesis of *H. hydrothermalis* Y2, based on a Placket Burman design experiment (data not shown). Furthermore, 100 g L^−1^ NaCl was found to be suitable for its ectoine production in the MMG medium. Conventionally, biological treatment is known to be strongly inhibited by high salinity and saline effluents are usually treated through the energy-consuming physico-chemical technology of high cost [41]. Therefore, the treatment of wastewater from ectoine fermentation greatly increases the cost and needs to be addressed for reducing salinity. In some previously pioneered works for ectoine production, the Ect cluster was also heterologous expressed in some hosts and achieved ectoine production under low-salt conditions, e.g., *Escherichia coli* [42] or *Corynebacterium glutamicum* [43]. Recently, a supreme productivity of 65 g L^−1^ was obtained in an engineered *C. glutamicum*, by using strategy of transcriptional balancing [44].

Based on our previous findings, the knockout of NhaD2, a Na^+^/H^+^ antiporter from strain Y2, seriously impaired the saline resistance at pH 10.0 whereas modest inhibition appeared at pH 8.0. Differently, the absence of Mrp was lethal to the saline resistance of the strain, with a completely inhibition at both pHs of 8.0 and 10.0 when confronting high salinity stress [27]. Explanations for these impaired saline resistance is that more Na^+^ was retained intracellular and toxic to the cells of those Na^+^/H^+^ antiporter deficiency. To alleviate the osmotic stress of Na^+^/H^+^ antiporter-deficient strain, we supposed that equal ectoine might be synthesized under a somewhat lower environmental salinity. Therefore, two major antiporters of NhaD2 and (or) Mrp was deleted and the corresponding mutants were tested for their abilities in ectoine production.

In agree with our previous observations, in the ectoine fermentation media of pH 9.0, the NhaD2-deficient strain showed minor influence to the growth and ectoine synthesis of Y2/*ΔectD*/*ΔdoeA*; while the Mrp-deficiency strain was sensitive to 100 g L^−1^ NaCl and more ectoine could be accumulated in the medium containing 80 g L^−1^ NaCl. Furthermore, the ectoine production of strain Y2, double mutant Y2/*ΔectD*/*ΔdoeA*, and Y2/*ΔectD*/*ΔdoeA/Δmrp* were compared in the auto-controlled bioreactors. In agreement with our speculation, under a reduced salinity, the Mrp-deficient strain synthesized an equal amount of ectoine to that of Y2/*ΔectD*/*ΔdoeA*. This confirmed the engineering feasibility of some halophilics in compatible solute production: weaken their Na^+^(K^+^) ions efflux ability and thereby lower the medium salinity for various compatible solute productions.

In the fed-batch bioreactor, the final NaCl concentration was set as 60 g L^−1^ and 50 g MSG was added to keep a constant content above 20 g L^−1^. After 48 h fermentation, the maximum ectoine titer was 10.5 g L^−1^, with a yield of 0.21 g g^−1^ MSG and a specific production of 765.0 mg (g CDW)^−1^. *Halomonas* species are the well-known strains for ectoine-production, including *H. salina* [45, 46], *H. elongate* [35], *H. boliviensis* [33], *H. ventosa* [47]. Besides these, we firstly demonstrated the functions of ectoine synthesis and catabolic pathways in strain *H. hydrothermalis* Y2 [48]. *H. elongate* is the most well-studied strain for the pathway in ectoine synthesis and metabolism [23]. These strains had also been reported for their ectoine productivities under various substrates and fermentation strategies. By using a fed-batch process, the intracellular ectoine in *H. elongate* reached to 15.9 g L^−1^ based on an optical intensity 92.9 (OD_600nm_) [35]. To our best knowledge, it is the highest production so far in the natural hosts. However, no specific production and productivity data available for this fermentation process. In combination with a two-step process, 6.0 g L^−1^ ectoine was accumulated in the cells of *H. boliviensis* DSM 15516^T^, after 9 h cultivation under 18.5% NaCl stress [33]. *H. salina* DSM5928^T^ is the only ectoine excreting strains reported so far. During a combined fermentation process of growing and resting cells, 14.86 g L^−1^ ectoine was accumulated with an excretion rate 61.6%. The productivity in a 10-L fermenter was 7.9 g L^−1^ d^−1^ and 0.14 g g^−1^ MSG [45]. Interestingly, strain *H. hydrothermalis* Y2 also possessed ability of ectione excretion and thus kept a constant concentration (around 4.0 g L^−1^) in the cells during the cultivation period of 18–48 h. This is well accordance with a conclusion revealed in *H. elongata* DSM2581, i.e., ectoine synthesis could be regulated to reach and maintain a constant cytoplasmic ectoine by using a putative export-uptake cycle [49].

## Conclusion

**Fig. 6:**
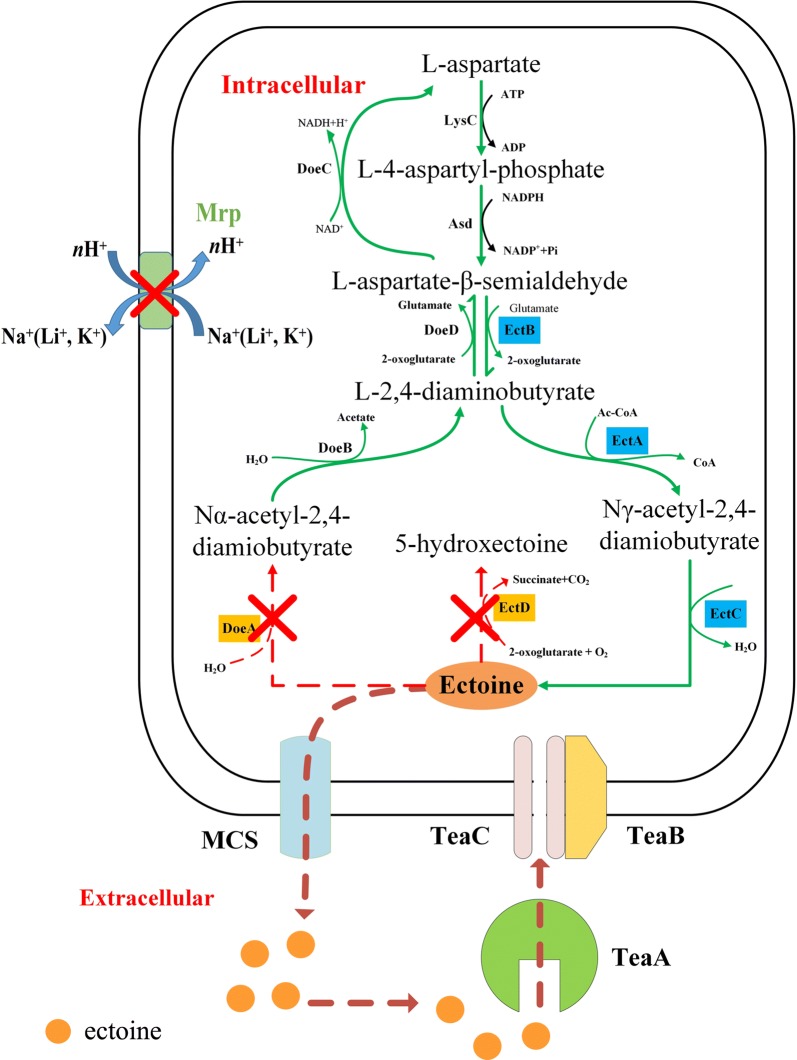
Diagram of ectoine metabolism in *H. hydrothermalis* Y2 and the disrupted pathways in this study. The disrupted pathway was shown as red cross, MSC means mechanic sensitive channel for ectoine efflux, TeaA, TeaB, and TeaC are proteins for ectoine uptake

Figure 6 shown the metabolic diagram of ectoine in strain *H. hydrothermalis* Y2 and the genetically engineering strategies adopted in this study. Two known pathways for ectoine catabolism were disrupted in which the Doe pathway showed to be much effective than EctD for ectoine accumulation. Furthermore, other unknown pathway for ectoine utilization was supposed and need to be further addressed. Based on the double mutant/*ΔectD*/*ΔdoeA*, the disruption of Mrp antiporter could alleviate the medium salinity for ectione synthesis and suggested potentials of this strategy for synthesizing some compatible solute under lower salinity environments.

## Methods

### Bacterial strains, plasmids, and culture conditions

The strains and plasmids used in this study are listed in Table 1. *H. hydrothermalis* Y2 was isolated from pulp mill wastewater (Previously *Halomonas* sp. Y2) and deposited in the China Center for Type Culture Collection (CCTCC No. M208188). The strain was routinely cultured in the Luria–Bertani (LB) medium containing (L^−1^) 10 g glucose, 10 g peptone, 5 g yeast extract, and 10 g NaCl, at pH 9.0. The standard ectoine was purchased from Sigma-Aldrich for HPLC analysis. MMG medium with the supplementations of indicated concentrations MSG, NaCl, and 500 mL L^−1^ inorganic salt mixture. To test the ectoine-producing capacity of mutants Y2/*ΔectD* and Y2/*ΔectD*/*ΔdoeA*, 34 g L^−1^ MSG and 100 g L^−1^ NaCl were supplemented into the MMG medium of pH 9.0. The inorganic salt mixture comprises (L^−1^) 3 g KH_2_PO_4_, 11.8 g K_2_HPO_4_, 0.4 g MgSO_4_·7H_2_O, and 0.01 g MnSO_4_·4H_2_O.

**Table 1:** Strains and plasmids used in this study

Strain or plasmid	Relevant phenotype or genotype	Reference
Strain		
*Halomonas hydrothermalis* Y2	Halotolerant alkaliphilic strain	[26]
*E. coli* DH5α	Competent cell for cloning	
*E. coli* S17-1	Competent cell for gene knockout	
Y2/Δ*ectD*	Strain Y2 with *ectD* deficient	This study
Y2/Δ*ectD/*Δ*doeA*	Strain Y2 with *ectD* and *doeA* deficient	This study
Y2/Δ*ectD/*Δ*doeA/*Δ*nhaD2*	Strain Y2 with *ectD*, *doeA*, and *nhaD2* deficient	This study
Y2/Δ*ectD/*Δ*doeA/*Δ*mrp*	Strain Y2 with *ectD*, *doeA*, and *mrp* deficient	This study
Y2/Δ*ectD/*Δ*doeA/*Δ*nhaD2/*Δ*mrp*	Strain Y2 with *ectD*, *doeA*, *nhaD2*, and *mrp* deficient	This study
Plasmid		
pEASYBlunt	Cloning vector	TransGen Biotech
pK18*mobSacB*	K^mr^ *mobsacB*	
pK18*mobSacB*/Δ*ectD*	pK18*mobSacB* with Δ*ectD*	This study
pK18*mobSacB*/Δ*ectD/*Δ*doeA*	pK18*mobSacB* with Δ*ectD* and Δ*doeA*	This study
pK18*mobSacB*/Δ*ectD/*Δ*doeA/*Δ*nhaD2*	pK18*mobSacB* with Δ*ectD*, Δ*doeA,* Δ*nhaD2*	This study
pK18*mobSacB*/Δ*ectD/*Δ*doeA/*Δ*mrp*	pK18*mobSacB* with Δ*ectD*, Δ*doeA,* Δ*mrp*	This study
pK18*mobSacB*/Δ*ectD/*Δ*doeA/*Δ*nhaD2/*Δ*mrp*	pK18*mobSacB* with Δ*ectD*, Δ*doeA,* Δ*nhaD2,* Δ*mrp,*	This study

### Genome analysis of ectoine metabolite pathway of *H. hydrothermalis* Y2

Sequences encoding ectoine metabolic enzymes were extracted from the genomic sequence (NCBI no: CP023656) of strain Y2 [48] and manually verified using BLAST searches against NCBI database and the UniProt. The metabolic pathway of ectoine (Fig. 1) was constructed according to studies previously published [17, 23, 50, 51].

### Gene knockout

After cultured in the LB medium at 30 °C for 10 h, cells of strain Y2 were collected by centrifugation at 4000×*g* for 10 min. Genomic DNA was isolated from the cell pellets using the ChargeSwitch^®^ gDNA Mini Bacteria Kit (Life Technologies) according to the manufacturer’s instructions. Recombinant PCR was applied to fulfill gene recombination from the desired genes and to form the defective gene fragments, using the primers listed in Table 2 and genomic DNA as the template. The recombinant PCR fragments, lacking 411 bp in *ectD* and 333 bp in *doeA*, were ligated into the shuttle vector pK18*mobsacB* and transferred into the strain *E. coli* S17-1. After co-cultivation of the recombinant *E. coli* S17-1 with *H. hydrothermalis* Y2 in LB medium, the recombinant strains were selected by LB medium with kanamycin (50 μg mL^−1^) and ampicillin (100 μg mL^−1^). Finally, the resulting mutants Y2/*ΔectD*/*ΔdoeA* were selected by LB medium containing 20% (w/v) sucrose at 37 °C and verified by PCR and DNA sequencing [27].

**Table 2:** Primers for gene knockout used in this study

Name	Sequence
*ectD*-F1	CGCGGATCCATGACAGTCGGCAATACACCTCTC
*ectD*-R1	AAGGCAGGGCACCCGCTCGCGGTAAGCAT
*ectD*-F2	TTACCGCGAGCGGGTGCCCTGCCTTGGTGAAA
*ectD*-R2	CCCAAGCTTTTAAACGCCCGGCTGCCAAG
*doeA*-F1	CGCGGATCCGTGGCCCAGTGGCGCGTTTCA
*doeA*-R1	TTGAACACCCCGCCGGTGTGCCAGCCAC
*doeA*-F2	TGGCACACCGGCGGGGTGTTCAAGCGCTA
*doeA*-R2	CCCAAGCTTCTACTTCACGAACAGCTG

The restriction sites in primers are underlined

For deletions of *mrp* and *nhaD2*, *E. coli* S17-1 strains carrying the recombinant plasmids [27] were co-cultivated with the double mutant Y2/*ΔectD*/*ΔdoeA*, and the mutants were selected as above described.

### Influence of NaCl concentration to the ectoine synthesis

Strains Y2, Y2/*ΔectD*/*ΔdoeA*, Y2/*ΔectD*/*ΔdoeA*/*Δmrp*, Y2/*ΔectD*/*ΔdoeA*/*ΔnhaD2*, and Y2/*ΔectD*/*ΔdoeA*/*Δmrp/ΔnhaD2*, were cultured in the MMG medium with various NaCl concentrations (20 to 100 g L^−1^). Samples were taken after 24 h cultivation, centrifuged and measured for the cell dry weight, as well as the intracellular and extracellular ectoine concentrations.

### Batch fermentation in 1-L fermenter

For seed preparation, the wild-type strain *H. hydrothermalis* Y2 and its mutants were cultured in the LB medium (with 10 g L^−1^ glucose supplementation) at 30 °C for 8 h. With same biomass inoculation, these strains were transferred into a 1-L Multifors fermenter (Infors HT) that containing 800 mL MMG medium. For strain Y2/*ΔectD*/*ΔdoeA*/*Δmrp*, the MMG medium contained 80 g L^−1^ NaCl while those for the other two strains was 100 g L^−1^ NaCl. During 48 h fermentation at 30 °C, the pH was kept constant at 9.0 with automated addition of 6 M HCl or NH_4_OH. By adjusting stirrer speed and aeration rate, dissolved oxygen was maintained at above 30% during the cultivation. During fermentation period, samples were taken at interval for measuring the dry weight and ectoine accumulation.

### Fed-batch fermentation of mutant Y2/*ΔectD*/*ΔdoeA/Δmrp*

Fed-batch fermentation was performed in the 1-L Multifors fermenter (Infors HT), with an initial NaCl concentration of 20 g L^−1^, glucose 5 g L^−1^, and MSG 20 g L^−1^. During fed-batch cultivations, the pH was maintained at 9.0 by using 6 M HCl or NH_4_OH. The dissolved oxygen was maintained at above 30% by adjusting the stirring speed manually. In the 72 h fermentation process, the concentration of MSG and glucose were detected by SBA-40D (Shandong academy of Sciences) every 3 h. During the first 24 h fermentation process, the MSG content in the bioreactor was kept at 20 (± 5) g L^−1^ by off-line analyzing of samples and feeding 50% MSG. The NaCl feeding solution was prepared and added at 12, 15, 18 h after inoculation, until reached to a final NaCl concentration of 60 g L^−1^.

### Analysis methods

Cell growth was monitored by sampling, centrifuging, and measuring the CDWs. For intracellular ectoine analysis, cells were collected by centrifuging at 6000×*g* for 10 min, washed twice with 1.5 M NaCl solution, resuspended into the distilled water, and passed through a French press (Aminco). After centrifugation at 8000×*g* for 10 min, two volumes ethanol were added to the supernant and the mixture were passed through a 0.25 μm filter for intracellular ectoine measurements. For extracellular ectoine measurement, the supernants were mixed with two volumes of ethanol and filtered as above described. The ectoine concentrations were quantified by a Shimadzu HPLC system (Kyoto, Japan) that equipped with a Venusil XBP NH2 column (4.6 × 250 mm) and a UV detector monitored at a wavelength of 204 nm. Analysis were carried out with acetonitrile/H_2_O (60:40, V/V) as the mobile phase and a flow rate of 0.5 mL min^−1^.

The mean consumption rate of ectoine in the secondary growth phase was estimated as

$$ \bar{r} = \frac{{E_{\text{max} } - E_{\text{last}} }}{{T_{\text{last}} - T_{\text{max} } }}, $$

where $E_{\text{max} }$ and $E_{\text{end}}$ are the maximal and last concentrations of E, $T_{\text{max} }$ and $T_{\text{last}}$ are the correspondent time.

## Data Availability

The datasets used and/or analyzed during the current study are available from the corresponding author on reasonable request.

## Abbreviations

Ectoine: 1,4,5,6-tetrahydro-2-methyl-4-pyrimidinecarboxylic acid
MSG: monosodium glutamate
MMG: modified monosodium glutamate medium

## References

[1] Oren A (2008). Microbial life at high salt concentrations: phylogenetic and metabolic diversity. Saline Systems..

[2] Pastor JM, Salvador M, Argandona M, Bernal V, Reina-Bueno M, Csonka LN, Iborra J, Vargas C, Nieto JJ, Cánovas M (2010). Ectoines in cell stress protection: uses and biotechnological production. Biotechnol Adv..

[3] Reshetnikov AS, Khmelenina VN, Mustakhimov II, Kalyuzhnaya M, Lidstrom M, Trotsenko YA (2011). Diversity and phylogeny of the ectoine biosynthesis genes in aerobic, moderately halophilic methylotrophic bacteria. Extremophiles.

[4] Reshetnikov AS, Khmelenina VN, Mustakhimov II, Trotsenko YA (2011). Genes and enzymes of ectoine biosynthesis in halotolerant methanotrophs. Methods Enzymol.

[5] Lippert K, Galinski EA (1992). Enzyme stabilization by ectoine-type compatible solutes: protection against heating, freezing and drying. Appl Microbiol Biotechnol.

[6] Louis P, Trüper HG, Galinski EA (1994). Survival of *Escherichia coli* during drying and storage in the presence of compatible solutes. Appl Microbiol Biotechnol.

[7] Krutmann J (2000). Ultraviolet aradiation-induced biological effects in human skin: relevance for photoaging and photodermatosis. J Dermatol Sci.

[8] Kanapathipillai M, Lentzen G, Sierks M, Park CB (2005). Ectoine and hydroxyectoine inhibit aggregation and neurotoxicity of Alzheimer’s beta-amyloid. FEBS Lett.

[9] Graf R, Anzali S, Buenger J, Pfluecker F, Driller H (2008). The multifunctional role of ectoine as a natural cell protectant. Clin Dermatol.

[10] Sydlik U, Gallitz I, Albrecht C, Abel J, Krutmann J, Unfried K (2009). The compatible solute ectoine protects against nanoparticle-induced neutrophilic lung inflammation. Am J Respir Crit CareMed..

[11] Abdel-Aziz H, Wadie W, Abdallah DM, Lentzen G, Khayyal MT (2013). Novel effects of ectoine, a bacteria-derived natural tetrahydropyrimidine, in experimental colitis. Phytomedicine.

[12] Vargas C, Jebbar M, Carrasco R, Blanco C, Calderon M, Iglesias-guerra F, Nieto JJ (2005). Ectoines as compatible solutes and carbon and energy sources for the halophilic bacterium *Chromohalobacter salexigens*. J Appl Microbiol.

[13] Reshetnikov AS, Khmelenina VN, Trotsenko YA (2006). Characterization of the ectoine biosynthesis genes of haloalkalotolerant obligate methanotroph *Methylomicrobium alcaliphilum* 20Z. Arch Microbiol.

[14] Zhu D, Liu J, Han R, Shen G, Long Q, Wei X, Liu D (2014). Identification and characterization of ectoine biosynthesis genes and heterologous expression of the *ectABC* gene cluster from *Halomonas* sp QHL1, a moderately halophilic bacterium isolated from Qinghai lake. J Microbiol..

[15] Widderich N, Czech L, Elling FJ, Könneke M, Stöveken N, Pittelkow M, Riclea R, Dickschat JS, Heider J, Bremer E (2016). Strangers in the archaeal world: osmostress-responsive biosynthesis of ectoine and hydroxyectoine by the marine *Thaumarchaeon nitrosopumilus maritimus*. Environ Microbiol.

[16] Louis P, Galinski EA (1997). Characterization of genes for the biosynthesis of the compatible solute ectoine from *Marinococcus halophilus* and osmoregulated expression in *Escherichia coli*. Microbiology.

[17] Ono H, Sawada K, Khunajakr N, Tao T, Yamamoto M, Hiramoto M, Shinmyo A, Takano M, Murooka Y (1999). Characterization of biosynthetic enzymes for ectoine as a compatible solute in a moderately halophilic eubacterium, *Halomonas elongata*. J Bacteriol..

[18] Garcia-Estepa R, Argandona M, Reina-Bueno M, Capote N, Iglesias-Guerra F, Nieto JJ, Vargas C (2006). The ectd gene, which is involved in the synthesis of the compatible solute hydroxyectoine, is essential for thermoprotection of the halophilic bacterium Chromohalobacter salexigens. J Bacteriol..

[19] Wohlfarth A, Severin J, Galinski EA (1990). The spectrum of compatible solutes in heterotrophic halophilic eubacteria of the family *Halomonadaceae*. J Gen Microbiol.

[20] Schulz A, Hermann L, Freibert SA, Hoffmann T, Heider J, Bremer E (2017). Transcriptional regulation of ectoine catabolism in response to multiple metabolic and environmental cues. Environ Microbiol.

[21] Jebbar M, SohnBösser L, Bremer E (2005). Ectoine-induced proteins in *Sinorhizobium meliloti* include an ectoine ABC-type transporter involved in osmoprotection and ectoine catabolism. J Bacteriol.

[22] Lecher J, Pittelkow M, Zobel S, Bönig T, Smits S, Schmitt L, Bremer E (2009). The crystal structure of UehA in complex with ectoine—a comparison with other TRAP-T binding proteins. J Mol Biol..

[23] Schwibbert K, Marin-Sanguino A, Bagyan I, Heidrich G, Lentzen G, Seitz H, Rampp M, Schuster SC, Klenk H-P, Pfeiffer F, Oesterhelt D, Kunte HJ (2010). A blueprint of ectoine metabolism from the genome of the industrial producer *Halomonas elongata* DSM 2581^T^. Environ Microbiol.

[24] Schulz A, Hermann L, Freibert SA, Bӧnig T, Hoffmann T, Riclea R, Dickschat JS, Heider J, Bremer E (2017). Transcriptional regulation of ectoine catabolism in response to multiple metabolic and environmental cues. Environ Microbiol.

[25] Schulz A, Stӧveken N, Binzen IM, Hoffmann T, Heider J, Bremer E (2017). Feeding on compatible solutes: a substrate-induced pathway for uptake and catabolism of ectoines and its genetic control by EnuR. Environ Microbiol.

[26] Yang C, Wang Z, Li Y, Niu Y, Du M, He X, Ma C, Tang H, Xu P (2010). Metabolic versatility of halotolerant and alkaliphilic strains of *Halomonas* isolated from alkaline black liquor. Bioresour Technol.

[27] Cheng B, Meng Y, Cui Y, Li C, Tao F, Yin H, Yang C, Xu P (2016). Alkaline response of a halotolerant alkaliphilic *Halomonas* strain and functional diversity of its Na^+^(K^+^)/H^+^ antiporters. J Biol Chem.

[28] Ryjenkov D, Tarutina M, Moskvin O, Gomelsky M (2005). Cyclic diguanylate is a ubiquitous signaling molecule in bacteria: insights into biochemistry of the GGDEF protein domain. J Bacteriol.

[29] Römling U, Galperin M, Gomelsky M (2013). Cyclic di-GMP: the first 25 years of a universal bacterial second messenger. Microbiol Mol Biol R..

[30] Zhang L, Lang Y, Nagata S (2009). Efficient production of ectoine using ectoine-excreting strain. Extremophiles.

[31] Bursy J, Pierik AJ, Pica N, Bremer E (2007). Osmotically induced synthesis of the compatible solute hydroxyectoine is mediated by an evolutionarily conserved ectoine hydroxylase. J Biol Chem.

[32] Kosuke T, Hideki N, Tsutomu T, Akihiko K (2013). Ectoine production from lignocellulosic biomass-derived sugars by engineered *Halomonas elongata*. Bioresour Technol.

[33] Van-thuoc D, Guzmán H, Thihang M, Hattikaul R (2010). Ectoine production by *Halomonas boliviensis*: optimization using response surface methodology. Mar Biotechnol.

[34] Van-thuoc D, Guzmán H, Quillaguamán J, Hattikaul R (2010). High productivity of ectoines by *Halomonas boliviensis* using a combined two-step fed-batch culture and milking process. J Biotechnol.

[35] Chen R, Zhu L, Lv L, Su Y, Li B, Qian J (2017). Optimization of the extraction and purification of the compatible solute ectoine from *Halomonas elongate*, in the laboratory experiment of a commercial production project. World J Microb Bio..

[36] Pastor J, Bernal V, Salvador M, Argandona M, Vargas C, Csonka L, Sevilla Á, Iborra J, Nieto J, Cánovas M (2013). Role of central metabolism in the osmoadaptation of the halophilic bacterium *Chromohalobacter salexigens*. J Biol Chem.

[37] KushwahaIndrani B, Narain J, Geethadevi V, Parashar D, Jadhav K (2019). Betaine accumulation suppresses the *de*-*novo* synthesis of ectoine at a low osmotic concentration in *Halomonas* sp SBS 10, a bacterium with broad salinity tolerance. Mol Biol Rep.

[38] Onraedt A, Walcarius B, Soetaert W, Vandamme E (2005). Optimization of ectoine synthesis through fed-batch fermentation of *Brevibacterium epidermis*. Biotechnol Prog.

[39] Saum S, Müller V (2008). Regulation of osmoadaptation in the moderate halophile *Halobacillus halophilus*: chloride, glutamate and switching osmolyte strategies. Saline Systems.

[40] Tao P, Li H, Yu Y, Gu J, Liu Y (2016). Ectoine and 5-hydroxyectoine accumulation in the halophile *Virgibacillus halodenitrificans* PDB-F2 in response to salt stress. Appl Microbiol Biotechnol.

[41] Lefebvre O, René M (2006). Treatment of organic pollution in industrial saline wastewater: a literature review. Water Res..

[42] Ning Y, Wu X, Zhang C, Xu Q, Chen N, Xie X (2016). Pathway construction and metabolic engineering for fermentative production of ectoine in *Escherichia coli*. Metab Eng.

[43] Pérez-García F, Ziert C, Risse JM, Wendisch VF (2017). Improved fermentative production of the compatible solute ectoine by *Corynebacterium glutamicum* from glucose and alternative carbon sources. J Biotechnol.

[44] Gießelmann G, Dietrich D, Jungmann L, Kohlstedt M, Jeon EJ, Yim SS, Sommer F, Zimmer D, Mühlhaus T, Schroda M, Jeong KJ, Becker J, Wittmann C (2019). Metabolic engineering of Corynebacterium glutamicum for high-level ectoine production: design, combinatorial assembly, and implementation of a transcriptionally balanced heterologous ectoine pathway. Biotechnol J..

[45] Lang Y, Bai L, Ren Y, Zhang L, Nagata S (2011). Production of ectoine through a combined process that uses both growing and resting cells of *Halomonas salina* DSM 5928T. Extremophiles.

[46] Chen W, Hsu C, Lan C, Chang Y, Wei Y (2018). Production and characterization of ectoine using a moderately halophilic strain *Halomonas salina* BCRC17875. J Biosci Bioeng.

[47] Zhu D, Niu L, Wang C, Nagata S (2007). Isolation and characterization of moderately halophilic bacterium *Halomonas ventosae* DL7 synthesizing ectoine as compatible solute. Ann Microbiol..

[48] Zhao Q, Meng Y, Li S, Lv P, Xu P, Yang C (2018). Genome sequence of *Halomonas hydrothermalis* Y2, an efficient ectoine-producer isolated from pulp mill wastewater. J Biotechnol.

[49] Grammann K, Volke A, Kunte HJ (2002). New type of osmoregulated solute transporter identified in halophilic members of the bacteria domain: TRAP transporter TeaABC mediates uptake of ectoine and hydroxyectoine in *Halomonas elongata* DSM 2581^T^. J Bacteriol.

[50] Peters P, Galinski EA, Trüper HG (1990). The biosynthesis of ectoine. FEMS Microbiol Lett.

[51] Göller K, Ofer A, Galinski EA (1998). Construction and characterization of an NaCl-sensitive mutant of *Halomonas elongata* impaired in ectoine biosynthesis. FEMS Microbiol Lett.

